# Predictive Factors of Development of Graves' Ophthalmopathy for Patients with Juvenile Graves' Disease

**DOI:** 10.1155/2016/8129497

**Published:** 2016-06-16

**Authors:** Dalia Jarusaitiene, Rasa Verkauskiene, Vytautas Jasinskas, Jurate Jankauskiene

**Affiliations:** ^1^Eye Clinic, Medical Academy, Lithuanian University of Health Sciences, A. Mickevičiaus 9, LT-44307 Kaunas, Lithuania; ^2^Institute of Endocrinology, Medical Academy, Lithuanian University of Health Sciences, Eiveniu 2, LT-50009 Kaunas, Lithuania

## Abstract

*Background*. Due to low incidence of Graves' ophthalmopathy (GO) among children, the manifestation is poorly analyzed, posing a risk to late identification of insidious disease.* Purposes*. To identify predictive factors that may influence the development of GO in pediatric and young patients with Graves' disease (GD).* Methods*. A cross-sectional study of patients newly diagnosed with pediatric or juvenile GD during 2002–2012 was conducted at the Hospital of Lithuanian University of Health Sciences. Ocular evaluation was based on European Group on Graves' Orbitopathy survey. The ocular manifestations were analyzed in relation to demographic, environmental, and clinical factors.* Results*. In total, 130 patients with juvenile GD were included; 29.2% had GO. Median age at GD onset was 17 yrs (IQR 4–29). Main symptoms of GO were eyelids retraction (73.7%), proptosis (65.8%), injection of conjunctiva (42.1%), and eyeball motility disturbance (21.1%). Major significant and independent risk factors for GO development were high initial concentration of FT4 (OR = 5.963), TTHAb (OR = 6.358), stress (OR = 6.030), and smoking (OR = 7.098).* Conclusion*. The major factors that could influence GO development were smoking, stress, and increased levels of initial TRAb, FT4. Slight proptosis, retraction of eyelids, and conjunctive injection were found as predominant ophthalmological symptoms in juvenile GO.

## 1. Introduction

Graves' disease (GD) is autoimmune disorder caused by autoantibodies that activate the thyrotropin receptor (TSH-R) and this results in stimulating thyroid gland to synthesize and secrete thyroid hormones [1]. GD affects the thyroid gland (diffusely enlarged goiter), the orbital and periorbital tissues (ophthalmopathy), and in rare cases pretibial skin or digits (dermatopathy or thyroid acropachy) [2–4].

GD is the most common cause of hyperthyroidism in children and adolescents [5–9]. Graves' disease accounts for 10–15% of all childhood thyroid diseases and is rare in subjects below 5 years of age [10, 11]. Some studies indicate that the incidence of juvenile GD is increasing [8, 12]. Juvenile GD as multifactorial disease is associated with a family history of autoimmune thyroid disease or other autoimmune disorders, environmental and hormonal factors, and the immune system disorders [13]. There is no single factor leading to clinical outcome of the disease [14]. However, the studies on risk factors show heterogeneous and diffuse findings.

As mentioned above, in some Graves' disease cases the ophthalmopathy occurs, which is called Graves' ophthalmopathy (GO). The incidence of GO in children is very low, about 1.7 to 3.5 cases per 100,000 population per year [15, 16]. The clinical manifestation of GO in children and adolescents is usually mild [17], self-limited, and occurring during the acute hyperthyroid state [18]. Severe ophthalmopathy in pediatric age is uncommon [15, 19, 20]. Due to low incidence of GO among children, the manifestation is poorly analyzed, mainly investigated only in adults [21, 22]. This poses a risk to identify the signs of insidious disease too late.

Results from psychological studies show that GO has negative impact on the quality of life and causes psychological disorders and emotional distress due to either predominant disfiguring eye signs or the cosmetics changes [21, 23, 24]. Nexo et al. (2014) study suggests that patients with Graves' disease have a great risk of work disability and unemployment [25].

With this study we aimed to evaluate the signs of juvenile Graves' ophthalmopathy and identify the predictive factors that may influence the development of Graves' ophthalmopathy in children and young adults with Graves' disease.

## 2. Material and Methods

We designed and conducted a cross-sectional study in 2014 at Department of Ophthalmology, Hospital of Lithuanian University of Health Sciences. The sample of the study is comprised of subjects enrolled from database of the Hospital of Lithuanian University of Health Sciences who have been diagnosed with incident of Graves' disease at the age below 30 years, during the period 2002–2012. Eligibility criteria for patients were as follows:

- Inclusion criteria: Graves' disease diagnosed at younger age than 30 years and absence of ophthalmological or systemic diseases.
- Exclusion criteria: patient being not available for evaluation and presence of other ophthalmological or systemic diseases.

The study protocol was approved by Kaunas Regional Biomedical Research Ethics Committee (No. BE-2-40, 2011). Every study subject or his/her parents or guardians (if subject was minor) have signed informed consent form.

The diagnosis of GD was made by endocrinologist and based on commonly accepted clinical and laboratory criteria: clinical symptoms of hyperthyroidism, increased concentrations of free thyroxin (FT4), free triiodothyronine (FT3), suppressed thyrotropin (TSH) concentrations, increased serum concentrations of TSH receptor antibodies (TRAb), and diffuse goiter on ultrasound. The following parameters from medical records of patients diagnosed with GD were recorded: age at GD onset, gender, concentrations of FT3, FT4, TTH, TRAb, antiperoxidase antibodies, (antiTPO) and antibodies against thyroglobulin (antiTg), goiter size by palpation, treatment, and ocular findings at the onset of disease. Physical examination of the thyroid was performed by endocrinologist and thyroid size was graded according to the criteria recommended by the World Health Organization [WHO].

Ophthalmological assessment was made based on European Group on Graves' Orbitopathy (EUGOGO) survey (2008). The following ophthalmological findings were recorded: best-corrected visual acuity using autophoropter “Nidek RT-3100” (Nidek, Japan) and optotypes projector “Nidek CP-770” (Nidek, Japan), slit-lamp investigation (TAKAGI SEIKO, SM-4N, Japan, 2008), intraocular pressure obtained by device “Auto Refractometer/Keratometer/Tonometer TONOREF II” (Nidek, Japan), proptosis measured by Hertel's exophthalmometry (in millimeters), monocular ductions (in degrees), eyelid signs (lid aperture, upper and lower lid retraction; normal upper eyelid position was considered to be 1–1.5 mm below superior limbus; normal lower eyelid position was considered to be at the level of inferior limbus), and indirect ophthalmoscopy (“Volk super 66 D lens,” Volk Optical, Inc.). The ocular manifestation was analyzed in relation to the thyroid disease course (remissions and recurrence episodes), clinical manifestation of disease, putative risk factors, family history of thyroid disorders, smoking habits, and duration of treatment of thyroid disease.

### 2.1. Statistical Analysis

Statistical analysis was performed using “SPSS for Windows” version 20.0 (SPSS Inc., Illinois, USA). The continuous variables were described as mean values ± standard deviation (SD) or median (with interquartile range IQR), while the categorical variables were described as numbers and percentages. The Kolmogorov-Smirnov test was used to test the normality of distribution of continuous variables and based on that the parametric or nonparametric tests were subsequently chosen. In bivariate analyses, the independent samples *t*-test or Mann-Whitney test was used for the comparison of continuous variables between two independent groups. For comparisons of categorical variables, chi-square or Fisher exact test was used. For two continuous indicators, the Spearman correlation was calculated. For evaluation of the effect of risk factors on Graves' ophthalmopathy development in Graves' disease patients, the univariate and multivariate linear regression analysis were conducted with odds ratios as effect estimates. The median values of biochemical results were chosen as cut-off point used in the analysis.

The level of statistical significance was set at *P* < 0.05 for all the tests.

## 3. Results

### 3.1. Study Sample

In total, 130 patients with juvenile GD were evaluated for signs of GO. For 38 (29.2%) ophthalmological signs of GO were present. General and disease characteristics of study sample (*n* = 130) are presented in Table 1.

82% of GO patients had first ophthalmological signs before the diagnosis of GD. After the GD diagnosis, the median time to development of GO was 1 month (minimum 0 and maximum 24 months). The diagnosis of juvenile Graves' ophthalmopathy was based on clinical symptoms and signs. The most common complaints were bulging eyes (76.3%), tearing (42.1%), and photophobia (31.6%). In our study, the GO was in active form in 21.1% of total GO cases; the clinical activity score (CAS) was ≥3 symptoms from maximum 7. The most frequent objective symptoms were eyelids (upper or/and lower) retraction 73.7%, slight proptosis (65.8%), and injection of conjunctiva 42.1%. Eye motility was limited in 21.1% of GO cases, but only in extreme gazes, corneal staining was found in 5.3%, while the optic neuropathy signs were not found.

### 3.2. Bivariate Risk Factor Analysis

We analyzed the associations of putative risk factors on the development of GO among GD patients (Table 2). Comparing probable biomedical risk factors for GD, we found significantly more frequent positive family history of thyroid disease in two generations of relatives in GO (+) group. The thyroid gland size in palpitation was found larger in GO (+) group. Other clinical risk factors did not influence the occurrence of GO. 5.6% (2) of Graves' disease patients smoked in adolescence (up to 18 years old) and 12.8% (12) older than 18 years. 21.5% (28) of patients mentioned that they felt physically stressful event in one-year period before GD signs were presented.

The median age at Graves' disease diagnosis of all patients was 17 (4–29) years, in pediatric group critical age for appearance of Graves' disease symptoms was 14 (12–16) years, and in young adults was 21 (19–24) years. Comparing the age of GD onset between GO (+) and GO (−) groups the statistically important difference was not found.

Analyzing the course of Graves' disease, we found that relapses of GD during first 2 years after the remission was achieved were significantly more frequent in GO (+) compared to GO (−) group 60.5% (23) versus 38.0% (35) (*P* = 0.019). The duration of first remission was longer than one year for 57.6% (54) in GO (−) and 28.9% (11) in GO (+) (*P* < 0.003).

Biochemical results at diagnosis are presented in Table 3. FT4, FT3, TRAb, and antiTPO concentrations at diagnosis were found to be significantly higher in the GO (+) compared to GO (−) group (Table 3).

### 3.3. Multivariate Risk Factor Analysis

For identification of independent risk factors for GO development in GD patients we conducted two-step logistic regression. In the first step we checked 12 putative factors that could influence the GO development. Univariate logistic regression analysis revealed higher TRAb concentration at the beginning of GD (OR = 10.00) and smoking (OR = 7.86). Detailed results from univariate regression analysis are presented in Table 4.

The multivariate regression analysis revealed four independent factors associated with GO development in GD patients, TRAb, FT4, stress, and smoking (*P* < 0.05) (Table 5). However, gender, FT3, and family history of thyroid disease cannot be regarded as independent risk factors for GO (*P* > 0.05). Of note, the strongest trigger for Graves' ophthalmopathy was smoking increasing the odds to develop the GO by more than 7 times. For comparison initial TRAb concentration 16 U/L and higher, stress, and FT4 concentration of 36 pmol/L and above were associated with almost 6 times higher odds to develop ophthalmopathy compared with absence of these risk factors.

## 4. Discussion

Graves' ophthalmopathy in young patients can not only affect visual function but also significantly impair the quality of life and lead to psychological and social problems due to manifestation of ocular symptoms that disfigure the appearance. Graves' disease appears typically between 40 and 60 years of age [1], while juvenile GD is relatively rare. There are many studies targeted to establish the risk factors for GO among adults, but younger age group has been underinvestigated and the impact on putative risk factors is less clear. It can be hypothesized that younger patients have an advantage because they have had less exposure to risk factors related to lifetime duration; however, similar GO prevalence rates among GD cases regardless of age pose this assumption under question.

The main findings of our study indicate that juvenile GO appeared from mild to moderate stages. In our study, eye symptoms among GD patients appeared mostly within one month from GD diagnosis (varying from 0 to 24 months). The manifestation of ophthalmopathy can predict the more aggressive course of Graves' disease. We found that elevated TRAb and FT4 titres at diagnosis of GD are useful predictive factors influencing the development of Graves' ophthalmopathy. Smoking and stress could work as a trigger in juvenile GO development.

### 4.1. Prevalence of Juvenile GD/GO

We found that Graves' ophthalmopathy developed in 29% of juvenile Graves' disease cases. This is quite similar with data of Krassas et al. who report that in Europe GO occurred in 33% of patients with juvenile Graves' hyperthyroidism [15]. It should be noted, however, that these estimates are lower than that found in other studies with ranging prevalence of 40–60% in juvenile GD patients [8, 12, 15, 17, 20, 26–35].

Previous research suggests that childhood GO is less severe and self-limited comparing to adulthood GO [15, 31, 36, 37]. Reasons for this GO clinical difference are still unclear.

### 4.2. Juvenile GO Signs and Symptoms (Clinical Aspects)

The findings on clinical manifestation of juvenile GO found in our study are consistent with previous research. It has been found that most of juvenile patients presented with mild exophthalmos, eyelids abnormalities such as lid lag, and lagophthalmos, where lower lid retraction was found most frequently [28, 29, 32, 33]. Nonetheless, even pediatric patients with GD require vigilance; in rare cases severe signs of GO such as limited extraocular motility and visual threatening complications due to corneal or optic nerve involvement were observed [26, 28, 31, 37–41]. Diana et al. (2014) and Holt et al. (2008) in their studies noted that the symptoms of GO in majority of prepubertal children are milder than in postpubertal children; the latter have such symptoms like restrictive strabismus, chemosis, or preorbital fat pad enlargement [29, 32].

Eyelid retraction and slight proptosis were the most predominant signs of GO in our study. Injection of conjunctiva and such symptoms as photophobia and tearing without corneal staining may be attributed to dry eye signs. Changes in ocular surface and tear film are very common in patients with thyroid disease [42–44]. Although dry eye syndrome in thyroid disorders is usually considered as a complication of autoimmune condition related to Graves' ophthalmopathy, there are several causes which could contribute to dry eye syndrome in those patients. Among such causes, lacrimal gland dysfunction due to autoimmune process, increased ocular surface exposure due to proptosis and eyelid retraction, and excess evaporation due to abnormally high tear film osmolarity may be mentioned [45–48].

### 4.3. GO as Predictor of Aggressive GD Form

The fact that the majority (82%) of GO patients had ophthalmological complaints before the GD was identified shows the importance of collaboration of pediatricians, ophthalmologists, and endocrinologists, since the ocular symptoms may indicate the thyroid disorders. In our study, eye symptoms among GD patients appeared mostly within one month from GD diagnosis (varying from 0 to 24 months). It suggests that both Graves' disease and Graves' ophthalmopathy are likely to develop simultaneously. Acuna et al. (2007) also have found the temporal relationship between diagnosis of thyroid dysfunction and the onset of ophthalmic signs; they showed that these two disorders vary between 0 and 6 months (2 weeks on average), with majority of patients having some ocular signs at the time of diagnosis [16]. An explanation for simultaneous beginning of GD and GO may be the same autoimmune processes in orbit and thyroid gland [18].

Our study revealed a strong association between GO symptoms and more aggressive autoimmune disease course: patients with GO also had more frequent relapses and shorter first remission period. Similar findings were described by Gogakos et al. (2010) and Anagnostis et al. (2013) and this may be useful to endocrinologists for prediction of the risk for more frequent relapses [37, 49]. In contrast, Quadbeck et al. (2005) interpreted that thyroid-associated ophthalmopathy was not associated with relapse rate [50].

### 4.4. Risk Factors for GO

Though Graves' ophthalmopathy was mentioned in medical literature in 1835 by Robert James Graves, the causal factors are still under clinical and scientific debates. The etiology of autoimmune diseases is multifactorial, involving the interaction of genetic, environmental, hormonal, and immunological factors playing the role in their development [51]. Nevertheless, the onset of at least 50% of autoimmune disorders has been attributed to unknown trigger factors [52].

We found that elevated TRAb and FT4 titres at diagnosis of GD are useful predictive factors influencing the development of Graves' ophthalmopathy. The TSH receptor (TSHR) is an established autoantigen in GD and may play an important pathogenic role as a useful marker to determine the activity and severity of GO as well [53–57].

It has been shown that TSHR expression is higher in GO orbital fat compared with normal orbital adipose tissue [53, 54, 58]; the TSHR is also expressed on the surface of lacrimal cells [45]. Acuna et al. (2007) found an association between positive TRAb levels and development of Graves' ophthalmopathy in children with Graves' disease, but they did not analyze the basal TSH, FT3, or FT4 concentration influence for GO development [16]. Also Diana et al. (2014) found markedly higher serum TSAb levels in the pediatric GD and GO sample compared to those with thyroid disease only [29].

TSHR antibodies are not linked to ophthalmopathy in all cases. It remains unclear why in some cases GO occurs many years after the development of Graves' hyperthyroidism or in patients with Hashimoto's thyroiditis in whom eye changes occur in the absence of TRAb production [59]. Therefore the results seem conflicting, showing that TRAb is not the main pathogenetic clue for development of GO.

Other thyroid antibodies, such as antiTPO, may also influence the extrathyroid manifestation, but the findings from previous studies are ambiguous. Diana et al. (2014) and Acuna et al. (2007) did not find significant association between antiTPO and GO or correlation between the levels of TRAb and antiperoxidase antibodies [16, 29]. In contrast, Besharati and Rastegar (2005) and Lee et al. (2014) found positive association between elevated antiTPO levels in children with GO [33, 60]. Khoo and associates found a significant correlation between negative antiperoxidase antibodies and Graves' ophthalmopathy in adult patients [61]. Wright-Pascoe et al. reported that both antiTPO and antiTg correlated with GO manifestation in adults [62].

In addition, thyroid hormones are also under debates regarding impact on GO. Majority (75–80%) of GO patients present Graves' disease as hyperthyroidism [63, 64], but GO may present in hypothyroid or even euthyroid status [65]. Lee et al. (2014) in retrospective study of 80 pediatric GD patients found that initial higher level of FT3 was associated with the presence of GO in childhood GD, but the initial serum levels of FT4 and TSH were not significantly different between the GD groups with and without GO [33]. Shibayama and colleagues (2005) did not find a relationship between concentration of FT3 and FT4 and Graves' ophthalmopathy [35]. The recent study by Stein et al. 2015 in large cohort of adult patients with newly diagnosed GD found that among risk factors the elevated TSH and thyroid stimulating antibodies (TSI) may negatively impact GO [66]. It was found that the risk of GO increases in case of elevated TSH level, using cut-off of 7 *μ*IU/mL.

In the present study we also tried to establish the trigger factors for juvenile GO development; one of them was cigarette smoking. Cigarette smoking as a potential risk factor for GO occurrence has been analyzed in majority of studies with conflicting results. Smoking is associated with an increased risk of development of signs of GO by 8 times (comparing with nonsmokers) [67–69]. Severe GO forms and lower effect of immunosuppressive treatment in smoking GO patients are more often observed compared to nonsmoking GO patients [70, 71]. One study found that the manifestations of GO begin to resemble the adult pattern in adolescence; this could be explained by increasing smoking prevalence in adults [26]. The risk of development of more severe GO signs as proptosis and diplopia is also correlated with the number of cigarettes smoked daily [14, 53, 67, 72]. All this was supported by Lee et al. (2010) who noted that smoking status was a predictive risk factor for a severe course of GO and the development of optic neuropathy [73]. The fact that there are less severe cases in children may explain that active smoking is unusual in children. However, Abraham-Nordling et al. (2011) and Nyström et al. (2013) were not able to find a correlation between smoking and GO development [74, 75]. Kampmann et al. (2014) in prospective study reported that smoking habits did not impact severity of GO [55].

There is an interesting theory that smoking might have direct irritative actions on the eyes accounting for inflammatory changes and modulation of immune reactions in the orbit. Smoking causes partial hypoxia, which stimulates the orbital fibroblasts to synthesize glycosaminoglycans which exacerbates extraocular muscles oedema and swelling [76].

Whether passive smoking may have the same impact as active smoking is not established; however, in a European survey of GO among children, the highest prevalence of pediatric GO was found in countries where the prevalence of smokers among teenagers was also highest: since more than 50% of children were less than 10 years of age, it is likely that passive smoking rather than active smoking influenced GO occurrence [15, 20].

In the current study we noticed that GO appeared more often in patients who mentioned that within last year before GD onset they had a stressful event. They listed such situations as learning and work stress (learning problems, dropping out from school or university, speaking in front of class and colleagues, conflicts with friends and loved ones, and loss of a job) and life stresses (death of close relatives, divorce of parents, financial problems, and traumas). The emotional stress has been long considered as possible etiology of Graves' disease, but the relationship between psychological factors and GO development is still not well known. In patients with Graves' disease, the stress scores correlated significantly with serum TSH receptor antibody activity and thyroid volume [52]. This hypothesis suggests the conclusion that stress is a possible trigger for an increased TSAb level which affects GO development.

Our study had some limitations. It comprised a small study sample due to low incidence of juvenile Graves' disease. In addition, we used a retrospective design for GD and GO incidence, which may also affect the findings of the study. The retrospective stage includes relatively long period and therefore we cannot exclude that some patients who were classified as having no GO might have GO signs at time of GD diagnosis. Further prospective studies are preferred to continue the assessment of relationship between thyroid disease course and extrathyroid manifestation. Nevertheless, as counterbalance to those limitations our study also had advantages, especially in terms of risk factor interactions, since younger patients have shorter exposure of negative factors.

## 5. Conclusion

Our findings clearly show that active smoking, stress, elevated initial TRAb, and FT4 levels played the major role in GO development for patients with juvenile Graves' disease. GO manifestation is associated with more aggressive course of GD in children and young patients. Slight proptosis, retraction of eyelids, and conjunctive injection were found as predominant ophthalmological symptoms in juvenile GO.

## Figures and Tables

**Table 1:** General and disease characteristics of study sample (*n* = 130).

Variable	GO(–) (*n* = 92)		GO(+) (*n* = 38)		*P* value
	Mean ± SD	Median (IQR)	Mean ± SD	Median (IQR)	
Age, years	23 (6.0)	24 (17–28)	22 (7.0)	23 (16–28)	0.468
Gender					
Female, % (*N*)	91.3 (84)		76.3 (29)		0.021
Male, % (*N*)	8.7 (8)		23.7 (9)		
Ethnicity					
Caucasian, % (*N*)	100 (130)				
BMI, kg/m^2^	20.68 (19.15–22.33)		20.76 (19.17–22.09)		0.938
Duration of GD, months	72 (8–122)				
Treatment, % (*N*)					
ATD MMI/PTU	54.6 (71)/4.6 (6)		22.3 (29)/2.3 (3)		0.916/0.720
Thyroidectomy	11.5 (15)		4.6 (6)		0.942
Radioactive iodine	2.3 (3)		1.5 (2)		0.589

GO (−): Graves' disease patients without Graves' ophthalmopathy; GO (+): Graves' disease patients with Graves' ophthalmopathy; BMI: body mass index; ATD: antithyroid drug; MMI: methimazole; PTU: propylthiouracil.

**Table 2:** Comparison of clinical findings and environmental risk factors between the groups with and without GO.

Factor	GO (−)	GO (+)	*P* value
Thyroid disease in family	38.0 (35)	68.4 (26)	0.002
Loss of weight	56.5 (52)	60.5 (23)	0.670
Drug-induced hypothyroidism	8.7 (8)	18.4 (7)	0.114
Thyroid gland volume (clinical assessment)			
I°	27.2 (25)	13.2 (5)	<0.001
II°	56.5 (52)	34.2 (13)	
III°	16.3 (15)	52.6 (20)	
Smoking	3.1 (4)	7.7 (10)	<0.001
Work character			
Does not work	11.5 (15)	3.8 (5)	0.761
Intellectual	51.5 (67)	23.1 (30)	
Physical	7.7 (10)	2.3 (3)	
Stress	10.0 (13)	11.5 (15)	<0.001

**Table 3:** Comparison of initial laboratory findings between the groups at GD onset.

	GO (−)	GO (+)	*P* value
FT4, pmol/L	32.40 (25.50–48.88)	59.19 (39.90–77.20)	<0.001
FT3, pmol/L	8.86 (6.80–14.77)	20.87 (11.57–28.80)	<0.001
TSH, mIU/L	0.01 (0.01–0.01)	0.01 (0.01–0.02)	0.162
TRAb, U/L	10.40 (4.90–22.00)	38.25 (23.58–61.43)	<0.001
AntiTPO, kU/L	68.00 (15.90–388.69)	146.00 (56.00–506.63)	0.045
AntiTg, kU/mL	92.50 (14.50–346.25)	39.30 (14.00–620.00)	0.641

**Table 4:** Risk factors for GO development in juvenile GD patients: univariate logistic regression.

Factor	Value	*β*	OR	95% CI		*P*
Gender	Female		1.000			
	Male	1.181	3.259	1.150	9.235	0.026

Smoking	No		1.000			
	Yes	2.061	7.857	2.285	27.017	0.001

Family history of thyroid disease	No		1.000			
	Yes	1.261	3.529	1.581	7.878	0.002

Drug-induced hypothyroidism	No		1.000			
	Yes	0.863	2.371	0.793	7.086	0.122

Stress before GD	No		1.000			
	Yes	1.377	3.963	1.651	9.516	0.002

FT4, pmol/L	0–35.99		1.000			
	36.00+	1.764	5.833	2.407	14.137	<0.001

FT3, pmol/L	0–11.99		1.000			
	12.00+	1.535	4.643	1.901	11.338	0.001

TSH, mU/L	0.020+		1.000			
	0–0.019	0.520	1.683	0.765	3.700	0.195

AntiTPO, kU/L	0–79		1.000			
	80+	0.526	1.692	0.773	3.706	0.188

TRAb, U/L	0–15.99		1.000			
	16.00+	2.303	10.000	3.487	28.679	<0.001

AntiTG, kU/L	0–79		1.000			
	80+	−0.251	0.778	0.230	2.628	0.686

Age at the diagnosis, years	0–17		1.000			
	18+	0.087	1.091	0.512	2.324	0.822

*β*: regression coefficient; OR: odds ratio; CI: confidence interval.

**Table 5:** Risk factors for GO development in juvenile GD patients: multivariate logistic regression.

Factor	*β*	OR	95% CI		*P*
Gender	1.530	4.620	0.949	22.502	0.058
Smoking	1.960	7.098	1.262	39.929	0.026
Stress	1.797	6.030	1.251	29.502	0.025
Family history of thyroid disease	0.736	2.145	0.627	7.341	0.224
FT4, pmol/L	1.786	5.963	1.366	26.025	0.018
FT3, pmol/L	0.020	1.020	0.285	3.656	0.975
TRAb, U/L	1.850	6.358	1.558	25.953	0.010

*β*: regression coefficient; OR: odds ratio; CI: confidence interval.
